# Inhibition of IgE Activity to Bind its High Affinity Receptor (FcεRIα) by Mouse Anti-IgE Cε3∼4 Monoclonal Antibody (QME5)

**Published:** 2009-12

**Authors:** Chun Xia Qiao, Ming Lv, Lei Ming Guo, Ming Yu, Yan Li, Zhou Lin, Xiao Li Hua, Chun Mei Hou, Jian Nan Feng, Bei Fen Shen

**Affiliations:** 1 *Institute of Basic Medical Sciences, Beijing, P. R. China;*; 2 *Medical Biotechnology Institute and Clinical Immunology Laboratory, Suzhou University, Suzhou, P. R. China*

**Keywords:** IgE, MAE11, computer-guided homology modeling, anti-IgE antibody, FcɛRIα

## Abstract

Using computer-guided homology modeling method, the 3-D structure of the Fv fragment of a functional anti-IgE antibody (MAE11) was constructed and the spatial structure of E24-MAE11 complex was modeled based on the crystal structure of IgE-Fc (*abbr.* E24) and molecular docking method. Then the identified epitope of IgE was determined theoretically, which showed the key role of IgE-Cɛ3 in interacting with both FcɛRIα and MAE11. By normal protocols, we immunized mice with purified protein E34 and screened six anti-E34 monoclonal antibodies. Purified antibodies could identify E34 by Western blot; furthermore, all of them could bind IgE by ELISA, in which QME5 seemed to be the best. Flow cytometry analysis displayed that only QME5 could bind membrane IgE and it could compete with membrane FcɛRIα to bind soluble IgE. Meanwhile, QME5 couldn’t bind FcɛRIα-attached IgE, which suggested no hypersensitivity in triggering the target cells (mast cells or basophils) by crosslinking or inducing the release of a variety of chemical mediators.

## INTRODUCTION

Immunoglobulin E (IgE) was the last of the immunoglobulins discovered by Ishizaka *et al* in 1966 and the least abundant human immunoglobulin class (nano- to micro-gram per micro-liter range in the serum of normal healthy individuals). IgE acts a key role in the allergic response and anaphylactic diseases such as asthma, allergic rhinitis, atopic dermatitis and food allergies. Unlike other immunoglobulin classes, IgE bind specifically and with a very high affinity to its receptor FcɛRIα on the surface of human basophils and mast cells (Ka=10^9^ M^−1^) (1); furthermore, the long half-life of IgE/FcɛRIα complex in *situ* (∼2 weeks, compared with only several hours for the comparable IgG complex) contributes to the permanent sensitization of target cells. IgE cross-linking of FcɛRIα^+^ cells by specific antigens results in the release of a variety of chemical mediators (*e.g*. histamine, leukotriene and prostaglandins) and cytokines, which show their effects by interacting with specific receptors on target organs (2). Recently, allergy or atopic diseases became a widespread and growing health problem. Current wide-used drugs such as antihistamines, corticosteroids and bronchodilators mainly alleviate allergic symptoms and concomitant inflammatory reactions without affecting the basic causes of the diseases.

Several strategies were mentioned to treat IgE-mediated allergic diseases by down-regulating IgE levels. The basic idea was that humanized anti-IgE antibodies could be used for the isotype-specific control of IgE (3). The anti-IgE antibodies must have a high affinity for IgE, and bind to membrane-bound IgE (mIgE) on mIgE-expressing B cells; meanwhile, they should not to bind FcɛRIα-attached IgE, nor to bind the low-affinity IgE-Fc receptors (FcɛRII, or CD23) (4). In 2003, a humanized anti-human IgE antibody, Omalizumab, was permitted by FDA to treat severe allergic diseases. Omalizumab could block FcɛRIα binding site on IgE and interfere the initiation of hypersensitive responses. It has been shown to be beneficial in the treatment of allergic diseases (5–7) with probably injection site reactions being the most commonly side-effects as reported adverse event in Omalizumab-treating people (8, 9), however, the incidence of anaphylaxis in clinical trials for Omalizumab was 0.1% (10). Although there have been some failure cases in Omalizumab monotherapy (11, 12), anti-IgE antibody seems eutherapeutic to most moderate or severe IgE-mediated allegic diseases by now.

Our previous work expressed and purified the truncated mutant IgE Cɛ3-4 (E34, aa330-547) in *E.coli* expression system (13) and IgE Cɛ2-4 (E24, aa224-547) in eukaryotic system mainly following the procedure described (14). For FcɛRIα alone couldn’t be located at the membrane with its own transmembrane domain, we truncated the transmembrane domain of Her2 at the C-terminus of the extracellular part of FcɛRIα in order to achieved the surface display of the receptor (15), then a stable cell line FI5F10 with extracellular FcɛRIα was established using CHO^dhfr-^ cells, by which novel anti-IgE antibodies could be evaluated easily. In this study we theoretically constructed the structure of E34 and the variable domains of anti-IgE monoclonal antibody MAE11 (parent antibody of Omalizumab) (16). And then the complex of E34 binding to MAE11 or FcɛRIα was modeled, by which it was considered that E34, which could be easily obtained from prokaryotic system as antigen, might replace IgE for neutralizing antibody preparation. After mice immunization, a non-anaphylactic anti-IgE antibody QME5 was screened, which had weak capacity of antagonizing membrane FcɛRIα to bind soluble IgE.

## MATERIALS AND METHODS

### Cells

Stable cell line FI5F10 with extracellular part of FcɛRIα was established using CHO cell line (CRL-2092) and conserved in our lab; SKO-007, a B lymphocyte cell line which was identified to express IgE (CRL-8033-1, Homo sapiens; IgE; lambda light chain) and SP2/0 (P3-X63-Ag8.653) were also conserved in our lab.

### Molecular Modeling

The heavy and light chain variable domains of MAE11 were constructed according to the canonical structures methods using the Swiss-PDB Viewer program (version 3.7) (http://www.expasy.org/spdbv/) (17) and the Swiss-Model automated modeling server at ExPASy (http://www.expasy.ch/). To ensure proper packing of the variable domains of the heavy chain (V_H_) and the light chain (V_L_) in the resulting models, the surface accessible solvent area and surface electrostatic potential of MAE11-V_H_ and MAE11-V_L_ were analyzed using InsightII 2005 software (MSI, 2005). Using molecular docking method, the 3-D structure of V_H_-V_L_ complex (Fv) was constructed. After structural optimization of Fv, the 3-D complex structure of MAE11-Fv and E34 was obtained with molecular docking method.

### ELISA

ELISA plates were coated at 4°C overnight. Then after being blocked with 1.5% BSA in PBS at 37°C for 1h, 100 μL specific protein (e.g. culture media supernatant) was added and incubated at 37°C for 1 h, followed by 100 μL HRP_conjugated polyclonal antibody for 45 minutes at room temperature (RT for short, the same below). The peroxidase reaction was developed with color development solution containing 5.5 mM *o*-phenylene-diamine hydrochloride (OPD) and 8.5 mM H_2_O_2_. The light absorbance was measured at 492 nm with ELISA reader.

### Flow cytometry

Cells at 5 × 10^5^ per sample were incubated separately with diluted ligand (E24 or E34 etc.) in 100 μL PBA (2% BSA, 0.1% sodium azide in PBS, pH 7.4) for 30 minutes at 4°C. FITC conjugated polyclonal antibody was added to determine the amount of membrane-binding ligand. Samples were analyzed on a Becton Dickinson FACSCAN flow cytometer.

### Immunization and hybridoma screening

Immunization and production of MAbs were carried out using standard protocols. Five 4-week-old female BALB/c mice were subcutaneously immunized with 100 μg purified truncated protein E34 in complete Freund’s adjuvant per animal. The animals were then boosted twice at 4-week intervals using 100 μg antigen in incomplete Freund’s adjuvant. Three days after the final booster using 100 μg antigen, one mouse was sacrificed, and its splenocytes were fused with NS-1 at a 5:1 ratio to a final concentration of 2 × 10^6^ cell/mL, and 200 μL cells was plated in each well on five 96-well plates. Hybrids were selected in RPMI 1640 Medium supplemented with 20% fetal calf serum and 5 × 10^−3^ M hypoxanthine, 2 × 10^−5^ M aminopterin and 8 × 10^−4^ M thymidine (HAT). After about 10 days, cell clones secreting antibodies against E34 were screened by ELISA, and the positive clones were selected and subcloned until all clones were positive.

### Production and Purification of MAbs

To produce the MAb in large quantity, 3 × 10^6^ hybridoma cells of all five clones were expanded and injected into the peritoneal cavity of mice. Usually, 5mL ascites was collected from each mouse. After 14 days, ascites were withdrawn and centrifuged at 1500 rpm for 5 minutes. The binding capacity of the ascites was analyzed by ELISA. The supernatant was collected and applied to a column of protein A-sepharose 4B which had been pre-equilibrated in PBS. The bound MAb was eluted with citric acid (pH 4.0) and dialyzed against PBS overnight. The purified proteins were analyzed by SDS-PAGE and Western Blot.

### Western Blot

E34 was mixed with SDS-PAGE loading buffer (5×, reduced). Proteins were separated with electrophoresis, and then they were transferred to a nitrocellulose sheet. After blocking with 5% skim milk at RT for 1 h, 10 μg/mL MAbs were added separately for 2 h at RT. 5 μg/mL HRP_GAM IgG (Biodesign) was used to display the specific E34 line by an enhanced chemilunimescence (ECL) method using ECL Western blotting detection reagents (Amersham Life Science). 5 μg/mL HRP_Omalizumab and A HRP_conjugated mouse anti-E34 monoclonal antibody (HRP_1095) were set as positive controls.

### Relative affinity constant of QME5 and IgE determined by ELISA (18)

To evaluate the affinity constant of QME5 and IgE, a modified ELISA method was used. In brief, diluted QME5 (from 4 to 0.0625 μg/mL) was added to IgE-coated plate, then after three washes, GAM_HRP was added for detecting the bound MAbs and OPD reaction system was used as described above. From the binding curve, 1 μg/mL QME5 was chosen to carry out the detection of affinity constant. QME5 and diluted IgE were mixed and incubated at 4°C overnight, and then the complexes were added to IgE-coated plate. Subsequent procedures were the same as described before. We used the following formula to calculate the affinity constant K,

$$\text{K}={\text{a}}_{0}\times (\frac{\text{A}}{{\text{A}}_{0}–\text{A}}–1)$$

Here, in certain condition of diluted antigen, a_0_ means the antigen concentration; A is the absorbance value in this specific condition; A_0_ means the absorbance value of positive control with no antigen in the pre-incubated complex. The mean value of all K values was the final affinity constant.

### Subclass of QME5

Mouse Mab Isotyping test kit (HBT, Cat. HL2020) was used to identify the subclass of QME5. 1 mL of the supernatant of QME5 was collected, freeze-dried and re-dissolved to a final volume of 100 μL. All procedures were carried out following the protocols.

## RESULTS

### Theoretical analysis of E34

**Figure 1:**
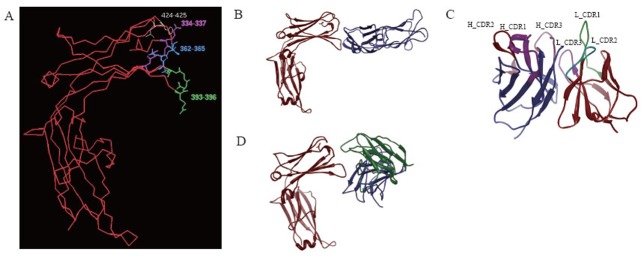
Theoretical analysis of E34. A, The identified key residues in E34 to bind FcɛRIα and MAE11; B, The 3-D crystal structure of E34/FcɛRIα complex, where the red ribbon denoted the main chain trace of E34, and the blue ribbon denoted the main chain of FcɛRIα; C, The optimized 3-D structure of MAE11 variable domain. The blue ribbon denoted the main chain trace of MAE11 V_H_, the red ribbon denoted the main chain trace of MAE11 V_L_; D, The optimized 3-D complex structure of E34 and MAE11, where the red ribbon denoted the main chain trace of E34, the blue denoted the main chain trace of MAE11 V_H_, and the green denoted the main chain trace of MAE11 V_L_.

Using Homology and Discover modules in InsightII 2005 software (MSI, 2005), the optimized 3-D structures of E34 (Fig. 1A) was obtained. According to the distance geometry method and interface analysis of IgE-Fc, weak intra-molecular interaction between Cɛ2 and E34 was determined. Four residues in E34 (i.e. Asp^330^, Ser^331^, Leu^429^ and Arg^427^) were involved in the van der waals interaction with Cɛ2, which indicated that lacking Cɛ2 domain might permit E34 to be more flexible. However, E34 keeps essential residues to exhibit the function of binding FcɛRIα or MAE11.

Besides, MAE11 variable domain (Fig. 1C) was also obtained using Homology and Discover modules, and then the complex of MAE11 and E34 (E34/MAE11) was modeled (Fig. 1D). Compared with the 3-D crystal structure of E34/FcɛRIα (Fig. 1B), the binding eptiopes in E34 recognized by MAE11 or FcɛRIα were determined to be located all in Cɛ3 domain; moreover, as shown in Table 1, the key residues of E34 to bind MAE11 were very close to that identified by FcɛRIα.

**Table 1:** Key residues in E34 to bind FcɛRI or MAE11

Key residues in E34	

FcɛRI	R^334^G^335^V^336^S^337^D^362^L^363^A^364^P^365^R^393^N^394^G^395^T^396^H^424^L^425^
MAE11	A^364^P^365^S^366^K^367^R^393^N^394^G^395^H^424^

### Binding activity of E34

**Figure 2:**
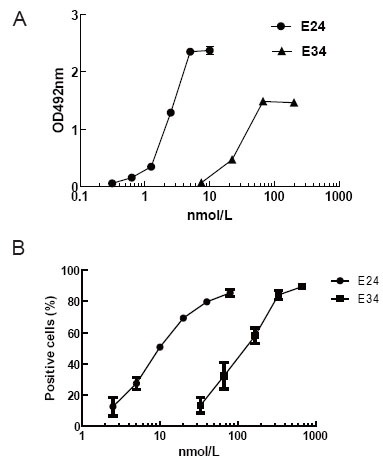
Binding activity analysis of E34 by ELISA and flow cytometry (FACS). A, E34 could bind Omalizumab in a dose-dependent manner by ELISA; B, E34 could bind membrane FcɛRIα on FI5F10 by FACS. The abscissa denoted the concentration of E24 or E34, while the y-axis denoted absorbance of OD492nm (A) or the percentage of positive cells (B).

ELISA plates were coated with 5 μg/mL Omalizumab, then diluted E24 or E34 were added, then samples were incubated 100 μL HRP_conjugated goat anti-mouse antibody (GAM_HRP) for 45 minutes at RT. As shown in Fig. 2A, similar to E24, E34 could detect Omalizumab in a dose-dependent manner. Furthermore, FI5F10 Cells were incubated separately with diluted E24 or E34. FITC_GAH IgE (FITC conjugated goat-anti-human IgE polyclonal antibody, Bethyl, Montgomery, Cat. N0. A80-108F) was added to determine the amount of membrane-bound ligand. FCM detection revealed that both two mutants and IgE had the capacity of binding the membrane-expressing receptor (Fig. 2B), indicating that the high-affinity receptor’s binding site located also in IgE Cɛ3∼4; besides, chimera E34 essentially retained the stereochemical structure of IgE Cɛ3-4 domain, which conformed to the results of computer aided design (CAD) above.

### Immunization and hybridoma screening

At 14 days of each immunization, blood was collected from mouse-tail and the titration of antibody against E34 was detected in serum. Usually, the titration was at least 1:10^−5^ before fusion. Hybrids were growing up in about 10 days and ready for screening. After ELISA analysis of the culture media, six positive cell clones (QME1, QME2, QME3, QME4, QME5 and QME6) were identified as secreting antibodies against E34 and further subcloned to generate stable hybridoma.

### Production, purification and identification of MAbs

**Figure 3:**
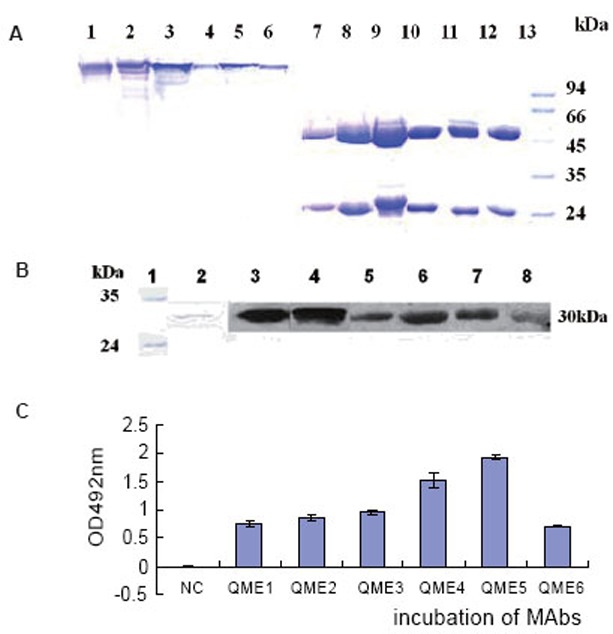
Purification and identification of six purified MAbs. A, SDS-PAGE analysis of six MAbs. 1∼6: nonreduced SDS-PAGE analysis of QME1∼QME6; 7∼12: reduced SDS-PAGE analysis of QME1∼QME6; B, Western Blot analysis of purified MAbs in E34 binding. 1: protein Mark; 2: SDS-PAGE of reduced E34; 3∼8: Western Blot analysis of QME1∼6 to recognize reduced antigen E34; C, ELISA analysis of six anti-E34 Mabs (QME1∼6) in binding human IgE.

The capacity of the ascites was analyzed by ELISAs, and the titration was about 1:10^−5^ (data not shown). Purified MAbs were analyzed by SDS-PAGE. As shown in Fig. 3A, two bands of about 25 and 50 kDa were generated under reduced conditions, while a single band of about 150 kDa was shown under nonreduced conditions as expected.

To test whether MAbs had the capacity of recognizing antigen, E34 protein was resolved on SDS-PAGE and then transferred to a nitrocellular membrane. Western blot analysis showed that all six MAbs at 10 μg/mL could recognize the antigen E34 (Fig. 3B); Furthermore, ELISA plates were coated with 1 μg/mL IgE. After being blocked with 1.5% BSA, 100 μL 2 μg/mL (∼13.3 nM) purified MAb were added separately, then samples were incubated with 100 μL GAM_HRP for 45 minutes at RT, which indicated that all of six MAbs could bind coated IgE as shown in Fig. 3C.

### Identification of QME5 in IgE binding

**Figure 4:**
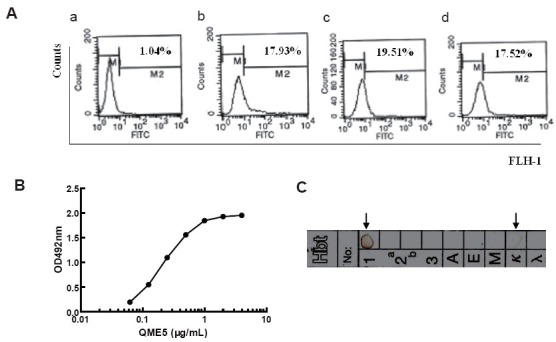
Identification of QME5 in IgE binding. A, QME5 could specifically recognize membrane IgE expressed on SKO-007 cell line by FACS. a: negative control (4C13); b: positive control (FITC_Omalizumab); c: positive control (FITC_GAH IgE); d: QME5; B, The standard curve of QME5 to bind IgE by ELISA. QME5 was diluted from 4 to 0.0625μg/mL. Arrow pointed was the chosen concentration of QME5 (1 μg/mL) for subsequent determination; C, The subclass identification of QME5 by Mouse Mab Isotyping test kit. Here displays the reaction card which presents the visible annulus (arrow pointed).

**QME5 could bind membrane IgE.** SKO-007 was incubated with 10 μg/mL QME5 for 30 minutes at 4°C, then FITC_GAM IgG was added to determine the amount of membrane-binding Mab. A mouse IgG1 Mab 4C13 (19) was set as negative control. FITC_GAH IgE and FITC_Omalizumab were set as positive controls. Flow cytometry analysis showed that the percentage of positive cells were 17.52% (Fig. 4A-d), which was similar to positive controls FITC_Omalizumab (17.93%, in Fig. 4A-b) and FITC_GAH IgE (19.51%, Fig. 4A-c). Compared with the negative sample mouse IgG1 4C13 (1.04%, Fig. 4A-a), we conclude that QME5 could specifically bind membrane IgE on SKO-007 cells. The other five MAb couldn’t bind membrane IgE even at the concentration of 50 μg/mL.

**Relative affinity constant of QME5 binding to IgE.** The standard curve shown in Fig. 4B indicated QME5 could bind coated IgE on a dose-dependent manner, by which 1 μg/mL QME5 was chosen as appropriate concentration (near the saturated point) for subsequent procedure of determining the relative affinity constant of QME5 using the formula mentioned above, and the calculated mean value is about 1.6 × 10^−7^ M, which is much weaker than that of FcɛRIα binding to IgE (10^9^ M^−1^).

### Subclass of QME5

After 24 hours’ incubation, QME5 interacted specifically with anti-IgG1 and anti-κ antibody on the reagent paper sheet, which formed a visible brown annulus (Fig. 4C), suggesting that the subclass of QME5 was IgG1, kappa light chain.

### QME5 didn’t have potential anaphylactic character

**Figure 5:**
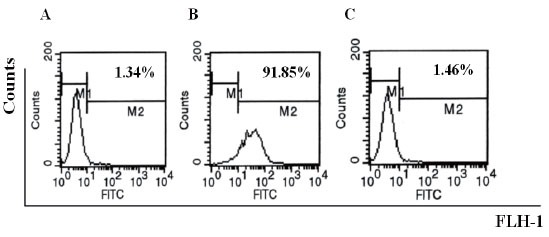
Flow cytometry analysis of QME5 in binding receptor-associated IgE. A, negative control (mouse IgG1 4C13); B, positive control (mouse anti-hIgE MAb 1095); C, QME5.

FI5F10 were incubated with 20 nM IgE, then after washing twice, cells were incubated with 75 nM QME5. FITC_GAM IgG was finally added in order to investigate the binding of QME5 and FcɛRIα-bound IgE. Cells incubated with 37.5 nM mouse IgG1 4C13 were set as negative control, and 37.5 nM mouse anti-IgE MAb 1095 was set as positive control. Results of this investigation are giving in Fig 5. that in contrast to the positive control 1095 (Fig. 5B), QME5 couldn’t bind FcɛRIα-bound IgE (1.46%, Fig. 5C), which was similar to the negative control (1.34%, Fig. 5A), indicating that QME5 had possibly no capability to trigger the cross-linking of IgE-loading FcɛRIα^+^ cells (basophils or mast cells) or to induce the release of a variety of chemical mediators and cytokines, although more functional experiments about QME5 are necessary in future study.

### QME5 could disturb the formation of IgE/FcɛRIα complex

**Figure 6:**
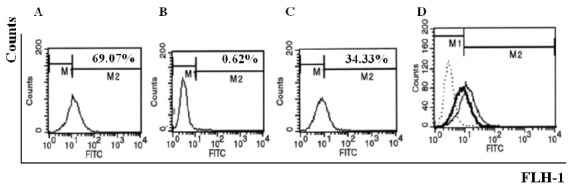
Flow cytometry analysis of QME5 in inhibiting IgE binding to FcɛRIα expressed on FI5F10 cells. A, positive control (IgE); B, negative control (preincubation of equimolar Omalizumab and hIgE); C, preincubation of QME5 and hIgE at the ratio of 100:1; D, superposition of A (IgE, normal line), B (Omalizumab, dashed line) and C (QME5, thick line).

750 nM QME5 and 7.5 nM IgE (molar ratio=1:100) were mixed and then the complex was added into FI5F10 cells, cells incubated with 7.5 nM IgE was set as a positive control, while equimolar Omalizumab pre-incubated with IgE was set as negative control. After washing, 5 μg/mL FITC_GAH IgE was added to determine the amount of membrane-binding IgE. Comparing to the positive control (69.07%, Fig. 6A), preincubation of IgE and QME5 could inhibit IgE molecular binding to its high affinity receptor FcɛRIα (Fig. 6C), which indicated that QME5 might have the capacity of neutralizing the soluble IgE against the receptor FcɛRIα *in vivo*.

## DISCUSSION

Interaction between human IgE and its high affinity receptor FcɛRIα on mast cells and basophils acts as important roles in initiating IgE mediated immune responses. IgE contains four constant domains (Cɛ1-Cɛ4). IgE-Fc (E24) contains Cɛ2-4 of IgE, in which Cɛ2 replaces the hinge region of IgG. E24 has the capacity of binding FcɛRIα with nearly the same affinity of IgE (14). The crystal structure of the human IgE-Fc/FcɛRIα complex to 3.5-Å resolution was reported in 2000 (20). It reveals that one receptor binds one dimeric IgE-Fc molecule asymmetrically through interactions at two sites, each involving one Cɛ3 domain of the IgE-Fc. The same as that in other members of the Fc receptor family, the interaction of one receptor with the IgE-Fc blocks the binding of a second receptor. Previous work presumed that the C-terminus two domains Cɛ3-Cɛ4 could bind FcɛRIα with the similar affinity with IgE.

There are four distinct regions within the IgE-Fc for FcɛRIα binding, including the Cɛ2-Cɛ3 linker (residues 334−336), the BC loop (362−365), the DE loop (393-396) and the FG loop (424−427). The 1:1 complex showed that three residues (P333, R334 and R427) involved in IgE Cɛ3 domains are observed as contact residues to bind FcɛRIα; besides, Y131 from the receptor projects into a pocket on the IgE-Fc formed by the BC and FG loops of Cɛ3 and the Cɛ2-Cɛ3 linker.

The extracellular part of FcɛRIα contains two domains (D1 and D2), in which the C-terminus D2 domain interacts directly with IgE. It contributes residues to bind IgE from three regions: the D1-D2 linker region (residue 85−87), the D2 BC loop (residues 110 and 113), and the D2 FG loop (residue 156-158). Eight residues including Y131 which located in the D2 domain of FcɛRIα interact directly with IgE; meanwhile residues from the D1 domain of the receptor do not form direct interactions with the IgE-Fc. It was also show that P426 from the IgE-Fc inserts receptor residues W87 and W110, forming a hydrophobic proline sandwich. All information above suggests new approaches with anti-IgE antibodies included to inhibit the binding of IgE to FcɛRIα for the treatment of allergic diseases.

Wurzburg (21) determined the 3-D structure of IgE-Fc through X-ray diffraction (2.3-Å) and showed a distinct bent of Cɛ3-Cɛ4 because of Cɛ2 domain contrasting to human IgG. It was considered that Cɛ2 domain does not affect the structure of E34 for rigid crystal structure showed that Cɛ2 (20) couldn’t bind directly to the receptor. Many investigations supported the crystal structure of IgE/FcɛRIα. Cɛ3 alone is the minimal units necessary for receptor binding (22, 23), and the monomer Cɛ3 had an affinity constant *K*a=5 × 10^6^ M^−1^ when binding to the receptor (22). Previous studies also testified that Cɛ2′-3′ (aa301-376) could bind the receptor, however, Cɛ3′-4 (aa340-547) which lacks Cɛ2-Cɛ3 linker (residues 334-336) domain has no binding capacity to its receptor (24). Therefore, considering the possible structure effect of Cɛ2-Cɛ3 linker (residues 334−336), we conjectured that Cɛ3-4 (E34, aa330-547) should have the capacity of receptor binding, which was similar to IgE-Fc fragment.

In this study, the 3-D structure of the functional anti-IgE antibody MAE11 Fv fragment was constructed using computer-guided homology modeling method. Based on 3-D crystal structure of IgE-Fc fragment (*abbr.* E24) and molecular docking method, the spatial structure of the interaction complex IgE (or E24)-MAE11 was modeled, and the identified epitope of IgE was determined theoretically, which showed that Cɛ3 in IgE was very important to interact with FcɛRIα and MAE11. Experiment results indicated that E34 could mainly retain the 3-D structure of IgE-Fc and the capacity to bind FcɛRIα or MAE11. According to our modeling results, the flexibility of E34 was possibly affected by lacking Cɛ2 domain, which might be a reason why E34 bound Omalizumab or membrane receptor FcɛRIα at a higher concentration (Fig. 2). The binding eptiopes in E34 identified by MAE11 were determined theoretically to be located mainly in Cɛ3 domain (Fig. 1C); meanwhile, according to 3-D crystal structure of E34/FcɛRIα complex (Fig. 1B), the key residues of E34 identified by MAE11 were superimposed on that identified by FcɛRIα. Therefore protein E34 purified from prokaryotic system (*E. coli*) could replace human IgE, which is scarce in blood serum or hard to be purified from cell supernatant, for preparing neutralizing anti-IgE antibodies target allergic diseases.

By normal protocols, we immunized mice and screened six anti-E34 monoclonal antibodies. Purified MAbs could identify the antigen E34 transferred to the nitrocellulose sheet by Western blot analysis. Then further detection of six MAbs by ELISA analysis showed that all of them could bind IgE molecule, in which QME5 (mouse IgG1 isotype) seemed to be the best (Fig. 3); meanwhile, flow cytometry analysis displayed that only QME5 had the capacity of binding membrane IgE on SKO-007 cell line (Fig. 4A). Moreover, QME5 couldn’t bind FcɛRIα-attached IgE, which suggested no hypersensitivity in triggering the target cells (mast cells or basophils) by cross-linking or inducing the release of a variety of chemical mediators (*eg*. histamine, leukotriene and prostaglandins) and cytokines (Fig. 5). The relative affinity constant of QME5 to bind IgE was about 1.6 × 10^−7^ M, which might be the reason why QME5 could compete with membrane FcɛRIα to bind soluble IgE only in a much higher dose (molar ratio of IgE: QME5 was 1:100) in order to hinder the formation of membrane IgE/FcɛRIα complex, for the affinity of IgE to bind its high affinity receptor FcɛRIα was about 10^−10^ ∼10^−11^ M (Fig. 6). Anyway, present QME5 might be more valuable for lab research, or diagnosis for human IgE detection, however, high-affinity or affinity-maturated anti-IgE antibodies screened from E34 immunized mice in future might be a candidate targeting IgE/FcɛRIα complex and should have potential capacity of neutralizing soluble IgE against the receptor *in vivo* for preventing or treating IgE-mediated allergic diseases.
